# Comparative Effects of Liraglutide-R-Alpha Lipoic Acid Combination and Donepezil on Diazepam-Induced Amnesia in Rats

**DOI:** 10.7759/cureus.87148

**Published:** 2025-07-02

**Authors:** Sonu Kumar, Hansraj Kumar, Shiv K Mudgal, Subodh Kumar, Harminder Singh

**Affiliations:** 1 Pharmacology and Therapeutics, Phulo Jhano Medical College and Hospital, Dumka, IND; 2 Pharmacology and Therapeutics, All India Institute of Medical Sciences, Deoghar, IND; 3 College of Nursing, All India Institute of Medical Sciences, Deoghar, IND

**Keywords:** amnesia, cognitive impairment, donepezil, liraglutide, r-alpha lipoic acid

## Abstract

Introduction: Cognitive impairment, including drug-induced amnesia, poses significant challenges in neurological research and therapy. Current treatments like donepezil provide limited benefits by targeting only cholinergic dysfunction. This study aimed to compare the cognitive-enhancing effects of the combination of liraglutide and R-alpha lipoic acid (R-ALA) with donepezil in a rat model of diazepam-induced amnesia.

Methods: Twenty adult Albino rats were randomized into four groups (n = 5 each): negative control, positive control (diazepam + saline), test (diazepam + liraglutide + R-ALA), and standard comparator (diazepam + donepezil). Diazepam (0.1 mg/kg, i.p.) induced amnesia, followed by treatments administered one hour before Morris Water Maze (MWM) trials. Escape latency times were recorded weekly at zero, one, two, three, and four hours post-treatment over five weeks. Data were analyzed by one-way ANOVA with Tukey’s HSD post hoc test (p < 0.05).

Results: The liraglutide-R-ALA group showed a significant and progressive reduction in escape latency compared to the positive control across all weeks and time points, with mean differences at the four-hour mark, ranging from 39.3 to 48.5 seconds (p < 0.001). Compared to donepezil, this combination showed statistically significant superiority at the third and fourth hours in weeks 1-5 (p-values ranging from 0.001 to 0.0001), with mean latency reductions of up to 20.65 seconds at four hours in week 5. Donepezil produced moderate improvements with higher variability and less consistent significance.

Conclusion: The liraglutide-R-ALA combination provides superior cognitive enhancement over donepezil in diazepam-induced amnesia by targeting multiple pathogenic mechanisms, highlighting the potential of multimodal therapies for cognitive impairment.

## Introduction

Cognitive impairment, particularly in the form of dementia, is a major global health concern, with Alzheimer’s disease (AD) representing the most common and debilitating form. Characterized by progressive memory loss, cognitive decline, and behavioral disturbances, AD affects more than 35 million individuals worldwide, a number projected to rise significantly in the coming decades [1]. The underlying pathology of AD involves the deposition of amyloid-beta (Aβ) plaques, formation of neurofibrillary tangles composed of hyperphosphorylated tau protein, cholinergic dysfunction, oxidative stress, and insulin resistance in the brain [2,3]. These multifactorial changes disrupt neuronal signaling and synaptic plasticity, particularly in the hippocampus, the brain region essential for learning and memory.

Despite several pharmacological interventions, current treatments for AD, such as donepezil (a reversible acetylcholinesterase inhibitor), offer only symptomatic relief without halting or reversing the underlying disease process [4]. Donepezil acts by enhancing cholinergic neurotransmission, but it fails to address key pathological contributors, such as oxidative stress and insulin signaling dysfunction [5]. Emerging therapeutic strategies are focusing on combination treatments targeting multiple mechanisms. In this context, glucagon-like peptide-1 receptor (GLP-1R) agonists, such as liraglutide, originally developed for the treatment of type 2 diabetes, have demonstrated neuroprotective properties, including the ability to enhance insulin signaling in the brain, reduce amyloid deposition, and promote neuronal survival [6,7]. R-Alpha lipoic acid (R-ALA), a potent antioxidant, has shown efficacy in reducing oxidative stress, enhancing acetylcholine synthesis, and protecting against Aβ-induced neurotoxicity [8,9].

Animal models are crucial in evaluating the efficacy of these novel agents. Diazepam, a benzodiazepine, is widely used to induce experimental amnesia in rodents by enhancing GABAergic inhibition, which results in impaired cognitive function and mimics some aspects of dementia [10]. The Morris Water Maze (MWM) is a well-validated behavioral test used to assess spatial learning and memory in rodents by measuring escape latency, the time taken to locate a hidden platform submerged in water [11].

This study was designed to compare the cognitive-enhancing effects of a combination of liraglutide and R-ALA with those of donepezil in diazepam-induced amnesic Albino rats using the MWM and evaluate whether combination therapy offers superior neuroprotective benefits and cognitive enhancement over standard treatments.

## Materials and methods

Study design

This randomized, controlled, laboratory-based experimental study was conducted over five weeks, employing repeated measures to track changes in learning and memory over time following the administration of different pharmacological agents.

Study population

The study was conducted on healthy adult Albino rats of either sex, weighing between 150 and 200 g. All animals were acclimatized to the laboratory environment for one week before the commencement of the experiment. The rats were maintained under standard conditions with a 12-hour light-dark cycle, controlled room temperature (22 ± 2°C), and humidity (55 ± 5%). They were provided a standard pellet diet and water ad libitum. All animals were handled daily to reduce stress during the experiment.

Inclusion and exclusion criteria

Rats included in the study were required to be healthy, active, and within the specified weight range. Animals showing signs of illness, lethargy, gross behavioral abnormalities, or unresponsiveness to handling were excluded. Additionally, rats that failed to demonstrate baseline navigational behavior during preliminary training in the MWM were also excluded to avoid bias in results due to pre-existing learning impairments.

Sampling and group allocation

A total of 20 rats were randomly divided into four groups (n = 5 per group) using a simple randomization technique. The group allocation was as follows: Group A (negative control), receiving normal saline (2 mL/kg) without diazepam; Group B (positive control), receiving diazepam (0.1 mg/kg, intraperitoneally), followed by normal saline (2 mL/kg); Group C (test group), receiving diazepam (0.1 mg/kg, i.p.), followed by a combination of liraglutide (0.1 mg/kg, subcutaneously) and R-ALA (5.5 mg/kg, i.p.); and Group D (standard comparator), receiving diazepam (0.1 mg/kg, i.p.), followed by donepezil (0.1 mg/kg, i.p.).

Drug administration was done one hour before each MWM trial to allow for optimal pharmacodynamic action.

Ethical considerations

Before the commencement of the study, ethical clearance was obtained from the Institutional Animal Ethics Committee (IAEC) of the Rajendra Institute of Medical Sciences, Ranchi (163/IAEC RIMS Ranchi/01/03/2021). The experiment strictly adhered to the guidelines set by the Committee for Control and Supervision of Experiments on Animals (CPCSEA), Government of India, with protocol number (1104/GO/Re/S/07/CPCSEA). All efforts were made to minimize animal suffering, including proper handling, gentle drug administration, and ensuring environmental enrichment. No animal was euthanized for this study. The present study forms an extension of the previously approved project. Specifically, the earlier publication focused on a subset of the experimental arms, whereas the current study explores an additional intervention group (liraglutide + R-ALA) for comparative evaluation. The study design, animal model, and protocol remained under the umbrella of the initially approved project [12].

Tools for data collection

Cognitive function was assessed using the MWM apparatus, a validated behavioral test for spatial learning and memory in rodents. The maze consisted of a large circular tank filled with opaque water, in which a hidden escape platform was submerged just below the water surface. Rats were subjected to weekly trials for five consecutive weeks. Escape latency time, defined as the time (in seconds) taken to locate the submerged platform, was recorded at zero, one, two, three, and four hours post-drug administration. A decrease in escape latency over time was interpreted as an improvement in memory and learning performance. Data were recorded manually using a stopwatch and observation sheet and verified through video recordings for accuracy.

Data analysis

Data obtained from each group were tabulated and expressed as mean ± standard deviation (SD). Intergroup comparisons of escape latency times were analyzed using one-way ANOVA, followed by Tukey’s Honestly Significant Difference (HSD) post hoc test to determine specific group differences. A p-value of less than 0.05 was considered statistically significant. Statistical analysis was performed using standard statistical software, such as IBM SPSS Statistics for Windows, Version 21.0 (Released 2012; IBM Corp., Armonk, NY, US).

## Results

The study involved four groups of rats (n = 5 each) to evaluate treatments for diazepam-induced amnesia. The negative control group received normal saline at 2 mL/kg without diazepam. The positive control group was administered diazepam at 0.1 mg/kg, followed by normal saline (2 mL/kg). The treatment group received diazepam (0.1 mg/kg) along with R-ALA at 5.5 mg/kg and liraglutide at 0.1 mg/kg. The standard treatment group received diazepam (0.1 mg/kg) plus donepezil at 0.1 mg/kg (Table 1). Groups A, B, and D were previously studied and published in an earlier article [12]. Their data are reused in this study to enable a comparative assessment of the new intervention (liraglutide + R-ALA combination) under similar experimental conditions.

**Table 1:** Details of drugs and doses administered to all groups of rats.

Groups	No. of rats	Diazepam	Drugs	Dose
Negative control (A)	5	Not applicable	Normal saline	2 mL/kg
Positive control (B)	5	0.1 mg/kg	Normal saline	2 mL/kg
R-Alpha lipoic acid and liraglutide (C)	5	0.1 mg/kg	R-Alpha lipoic acid and liraglutide	5.5 mg/kg and 0.1 mg/kg
Donepezil (D)	5	0.1 mg/kg	Donepezil	0.1 mg/kg

Performance of Group C (liraglutide + R-ALA)

**Figure 1:**
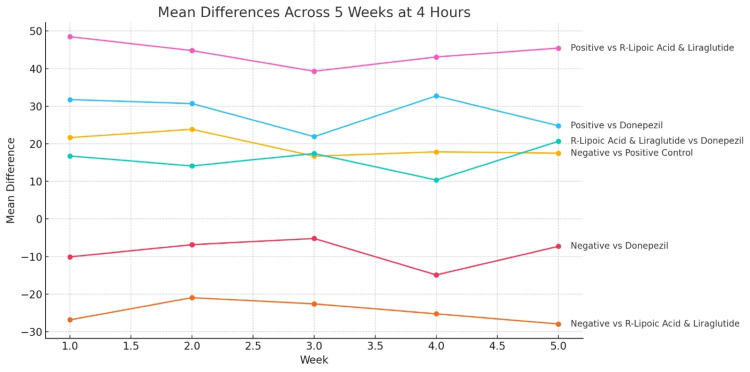
Mean escape latency over five weeks at different time intervals for Groups C and D

Group C rats demonstrated a consistent and progressive improvement in escape latency times across the five-week study period. At the two-hour mark, mean latency dropped from 40.4 ± 6.48 seconds in the first week to 32.5 ± 5.67 seconds by the fifth week. Similarly, the four-hour latency improved from 12.55 ± 2.28 seconds to 11.9 ± 1.47 seconds over the same period, suggesting significant retention of spatial memory even several hours after drug administration (Figure 1).

Performance of Group D (donepezil)

Group D rats, treated with donepezil, also exhibited improvement in escape latency times, though the effect was relatively modest when compared to Group C. At the two-hour time point, latency decreased from 46.8 ± 10.97 seconds in the first week to 44.3 ± 12.13 seconds in the fifth week. At four hours, the mean latency reduced from 29.3 ± 6.2 seconds to 32.55 ± 8.14 seconds, with some variability noted in week-to-week trends (Figure 1).

Statistical comparison between Group B (positive control) and Group C (test group)

Statistical analysis using one-way ANOVA followed by Tukey’s HSD test showed highly significant differences between Group C and Group B. At the second, third, and fourth hours of testing in all five weeks, Group C consistently outperformed Group B. For example, in the first week, the difference in mean latency between the groups at four hours was 48.5 seconds (p = 0.000). This trend continued across subsequent weeks, with mean differences at the four-hour mark, ranging from 39.3 to 45.45 seconds, all statistically significant at p < 0.001 (Table 2).

**Table 2:** Mean differences in escape latency (in seconds) between Groups B and C using one-way ANOVA, followed by Tukey’s HSD post hoc test HSD: Honestly Significant Difference.

No. of weeks	Second hour		Third hour		Fourth hour	
	Mean difference	p-value	Mean difference	p-value	Mean difference	p-value
First week	24.95	0.000	42.80	0.000	48.50	0.000
Second week	30.30	0.001	43.20	0.000	44.80	0.000
Third week	22.55	0.002	32.10	0.000	39.30	0.000
Fourth week	23.80	0.004	37.80	0.000	43.10	0.000
Fifth week	25.40	0.001	40.80	0.000	45.45	0.000

Statistical comparison between Group C (test group) and Group D (donepezil)

When comparing Group C and Group D, differences in escape latency were statistically significant at later time points, especially during the third and fourth hours of weekly testing. While the two-hour differences between the two groups were not statistically significant in the early weeks (e.g., week 1: mean difference = 6.4 seconds, p = 0.597), significant differences emerged in the third and fourth hours (week 1: 16.75 seconds at four hours, p = 0.001). By the fifth week, the superiority of the liraglutide-R-ALA combination was evident, with a mean difference of 20.65 seconds at the four-hour mark (p = 0.000) (Table 3).

**Table 3:** Mean differences in escape latency (in seconds) between Groups C and D using one-way ANOVA, followed by Tukey’s HSD post hoc test HSD: Honestly Significant Difference.

No. of weeks	Second hour		Third hour		Fourth hour	
	Mean difference	p-value	Mean difference	p-value	Mean difference	p-value
First week	6.40	0.597	19.50	0.001	16.75	0.001
Second week	7.30	0.843	11.20	0.069	14.10	0.001
Third week	9.80	0.380	9.90	0.258	17.40	0.000
Fourth week	2.20	0.999	13.60	0.036	10.35	0.001
Fifth week	11.80	0.224	18.25	0.001	20.65	0.000

## Discussion

This study demonstrates that the combination of liraglutide and R-ALA outperforms donepezil in reversing diazepam-induced memory impairment. The superiority of the liraglutide-R-ALA combination is likely due to its dual-action mechanism targeting key pathological features of cognitive dysfunction (oxidative stress and impaired insulin signaling), unlike donepezil, which targets only cholinergic transmission.

Liraglutide acts on central GLP-1 receptors, enhancing insulin-signaling pathways, reducing neuroinflammation, and promoting hippocampal neurogenesis and synaptic plasticity [6,7]. These actions are particularly relevant because insulin resistance in the brain is now recognized as a critical contributor to AD pathogenesis [13]. By reversing insulin resistance, liraglutide restores neuronal metabolism and supports long-term potentiation (LTP), a synaptic mechanism essential for memory formation.

R-ALA, on the other hand, is a potent mitochondrial antioxidant that neutralizes free radicals, maintains redox balance, and boosts acetylcholine synthesis [8,9]. Its capacity to mitigate oxidative stress complements liraglutide’s insulin-sensitizing effects. Together, they provide a comprehensive neuroprotective effect that extends beyond the narrow cholinergic focus of donepezil.

The consistent improvement in escape latency, particularly at the three- and four-hour intervals, suggests prolonged action of the combination therapy and better memory retention. These improvements were not only statistically significant but also showed low intra-group variability, indicating consistent treatment effects across subjects. In contrast, the donepezil group showed moderate and inconsistent results with higher standard deviations, indicating variability in responsiveness, similar to what is observed clinically [4,14].

The use of diazepam-induced amnesia offers a valid model for GABA-mediated cognitive suppression, simulating certain features of dementia such as slowed information processing and impaired learning [10]. Combined with the MWM, which is a gold-standard behavioral test for spatial memory, this model provides a reliable and translational platform for screening cognitive enhancers.

Furthermore, these results support the emerging consensus that multi-modal therapies may be more effective than monotherapies in complex disorders like AD. Most drug development efforts that targeted a single pathological pathway have failed in clinical trials. Agents like liraglutide and R-ALA, which address both metabolic and oxidative dimensions of neurodegeneration, hold greater translational promise [15,16].

From a translational perspective, both liraglutide and R-ALA have favorable safety profiles in humans and are already approved for other indications. This enhances the feasibility of rapid clinical translation for use in early-stage cognitive impairment or as adjuncts to existing therapies.

The study employed a randomized controlled design with validated behavioral tools. Multiple time-point assessments each week allowed for a nuanced understanding of drug action kinetics. The use of combination therapy targeting complementary mechanisms reflects a modern, pathophysiology-driven approach to neurodegeneration.

This study has several limitations. The small sample size (n = 5 per group) limits the statistical power and generalizability of the findings. Only behavioral outcomes (escape latency in MWM) were assessed without accompanying biochemical or histopathological analyses to validate the neuroprotective effects. Locomotor activity was not independently evaluated, which may have influenced maze performance. Additionally, sex-based differences were not explored, and the long-term effects of the interventions remain unassessed. Future studies with larger sample sizes, biomarker assessments, and prolonged follow-up are warranted.

## Conclusions

This study provides compelling evidence that liraglutide-R-ALA combination therapy offers superior cognitive improvement over donepezil in a rodent model of diazepam-induced amnesia. By targeting both insulin resistance and oxidative stress, the combination addresses multiple facets of neurodegeneration, offering a promising direction for future therapeutic strategies. Further studies involving biochemical markers, histology, and larger sample sizes are warranted to confirm these findings and assess clinical applicability.
